# SOURCES SOCIAL SUPPORT AND SUBTYPES OF ELDER MISTREATMENT AMONG CHINESE OLDER ADULTS: FINDINGS FROM THE PINE STUDY

**DOI:** 10.1093/geroni/igz038.2795

**Published:** 2019-11-08

**Authors:** Shenglin Zheng, Stephanie Bergren, XinQi Dong

**Affiliations:** Rutgers University, New Brunswick, New Jersey, United States

## Abstract

To examine the relationships between positive social support (PSS) and negative social support (NSS) from different sources and subtypes of EM, we used the data from a representative sample of 3,157 Chinese older adults aged 60 years or older in Chicago. Subtypes of EM include psychological mistreatment, physical mistreatment, financial exploitation, and caregiver neglect. Higher PSS from spouse and family members were less associated with lower likelihood to experience any of four self-reported EA subtypes. Higher PSS from friends was associated with lower likelihood of caregiver neglect. Increased levels of NSS from spouse and family members were associated with higher likelihood of psychological mistreatment, financial exploitation, and caregiver neglect. A significant association was also found between NSS from friends and psychological mistreatment. This study demonstrates the positive and negative aspects of social contexts in relationship to EM. Future longitudinal studies are needed to examine causal relationships.

